# Smartphone apps pertaining to aquaculture sector in Bangladesh: Current status and future potentials

**DOI:** 10.1016/j.heliyon.2024.e39396

**Published:** 2024-10-15

**Authors:** Diponkor Adikari, Fatema Jannat Munny, Debasish Pandit, Md Abu Kawsar

**Affiliations:** a Department of Aquatic Resource Management, Sylhet Agricultural University, Sylhet, 3100, Bangladesh; b Department of Aquaculture, Sylhet Agricultural University, Sylhet, 3100, Bangladesh; c Department of Fishery Resources Conservation and Management, Faculty of Fisheries and Ocean Sciences, Khulna Agricultural University, Khulna, 9100, Bangladesh

**Keywords:** Smartphone apps, Aquaculture, Fisheries, Production, Fish disease and treatment

## Abstract

In recent years, the aquaculture sector in Bangladesh has experienced significant technological advancements, notably the adoption of smartphone applications. This empirical study evaluates the current status of smartphone apps available in the aquaculture industry of the country using data obtained from the internet and field surveys. The study identified 41 smartphone apps currently used in the sector, where 58.53 % are related to pond-based aquaculture, 14.63 % are designed for biofloc system, 4.87 % pertain to cage aquaculture and 4.87 % marketing-aided corporate-related apps, 9.75 % focus on disease and treatment-related, and 7.31 % fall into miscellaneous categories. The “Poultry Animals and Aqua Index (PAAI)" app, which addresses aquaculture pharmaceutical inputs and fish disease treatment guidelines, emerged as the most downloaded app in this domain. Pearson correlation analysis indicated that app size or user ratings did not influence app downloads. The study also found that approximately 57.66 % of fish farmers are aware of these smartphone apps. Private fisheries professionals were the main driving forces behind the use of these apps, accounting for 52 % of the information spread. Despite mixed levels of satisfaction among farmers, about 25 % expressed a positive experience. Most of the farmers (57.66 %) acknowledged the awareness and beneficial impacts of using these apps in aquaculture. This study enhances the understanding of smartphone apps usage in Bangladesh to promote the aquaculture industry, highlighting its potential to promote industry growth, alleviate poverty, and contribute to economic development.

## Highlights

• Technological advancements have increased the number of aquaculture-based smartphone apps in Bangladesh.
• Fisheries professionals, feed sellers, and local farmers are driving fish farmers to use these apps.
• Most aquaculture-based smartphone apps in Bangladesh focus on pond-based aquaculture.
• Satisfaction levels vary among farmers using aquaculture apps, with notable concerns over feature specificity.

## Graphical abstract

**Figure:**
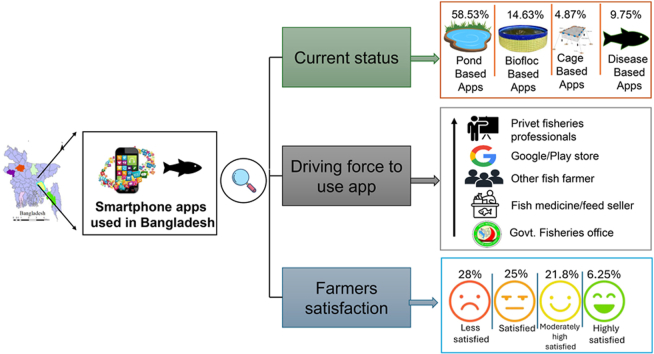

## 1 Introduction

The fisheries sector is essential for meeting the animal protein needs of the world's growing population [1]. Aquatic organisms are a global delicacy and a significant source of dietary protein. World aquatic animal production in 2020 had increased significantly, by more than 60 % compared to the average production levels seen during the 1990s, primarily due to global aquaculture production reaching a record 122.6 million metric tons (MT) [2]. Asian countries, including China, India, Vietnam, Indonesia, and Bangladesh, accounted for 91.6 % of this production [2]. Capture fisheries are declining due to several causes, including climate change, habitat loss, pollution and overfishing, making aquaculture a critical sector for supplying nutrition, especially in developing Asian nations [[3], [4], [5]].

Bangladesh, situated at the northern tip of the Bay of Bengal and surrounded by river systems, huge inland waters, and a vast coastal area, is well-positioned for successful fisheries and aquaculture practices [6,7]. With abundant resources, Bangladesh is ranked fifth among the world's top aquaculture-producing and self-sufficient fish-producing countries [8]. Freshwater fish are a common and affordable protein source in the country, with many people involved in this sector at various stages. More than 12 % of the Bangladeshi population, particularly in rural and coastal areas, are involved in aquaculture and fisheries for their livelihoods [8]. The expansion of aquaculture can ensure food security and provide employment opportunities for underserved and impoverished populations in Bangladesh [9].

Numerous conventional and modern technologies and equipment are used in different steps in aquaculture operations to enhance farm productivity [10,11]. These include dissolved oxygen meters, TDS meters, ammonia test kits, nitrite test kits, pH meters, feeding machines, conventional feed makers, electric aerators, electric blowers, lights, generators, boats, nets, etc. [12]. Recently, a wave of Internet of Things (IoT)-based technologies has emerged to revolutionize aquaculture, i.e., automatic feeding machines [11], sensor-based water quality monitoring [13], and closed-circuit video cameras for farm surveillance [14]. Additionally, other modern culture techniques, i.e., recirculatory aquaculture systems, aquaponics, and biofloc technology, have steadily gained popularity over the years [15].

Both the smartphone app and IoT industries are experiencing growth with the advent of innovative technology. The number of smartphone apps on the market is expanding, proving to be a substantial source of revenue for the IT industry [16]. Beyond being merely a piece of hardware, smartphones have ushered in a new era for software developers worldwide. Mobile learning apps were more effective in enhancing end-user knowledge compared to traditional teaching methods, due to the flexibility offered by mobile devices in terms of time and location [17,18]. Numerous ICT-aided projects have attempted to provide extension support; however, their impact has been limited by their narrow focus and restricted geographical reach [19,20]. Nonetheless, the development of mobile networks with higher data speeds and better connectivity, even in remote areas, along with the decreasing costs of mobile handsets globally, has driven the rapid growth of mobile applications. These advancements have helped bridge communication gaps, supporting research, extension services, farmers, and market integration [21]. Mobile phone-based information pathways can address the significant barrier of accessing farm advisory services, thus boosting farm productivity among small stakeholders [22,23]. Additionally, mobile applications have facilitated two-way information exchange [23], provided customized advisories to farmers, reduced information asymmetry, and improved knowledge levels among farmers [[24], [25], [26]].

Aquaculture is crucial for meeting the global demand for aquatic food, but recently this sector has faced challenges in fish growth and health monitoring. In recent times, AI advancements have offered solutions to optimize fish farming practices and ensure sustainability. AI technologies like machine learning and computer vision analyze large data volumes from fish farms, providing insights into growth patterns, feeding behavior, and health [27]. Smart monitoring systems use sensors and data analytics to track water quality and fish behavior, enabling timely interventions [28]. AI also optimizes feed management and reduces waste, while image analysis helps in early disease detection and targeted treatment [29]. Integrating AI in aquaculture enhances productivity, reduces environmental impact, and improves fish welfare [27].

Smartphone apps play a crucial role in disseminating essential information in the proper format and at the right time, significantly reducing the cost of communication and information [22]. By adopting smartphone apps, fish farmers can modify their culture techniques, administer treatments if fish are affected by diseases, and take preventive measures by observing fish conditions, all without needing to call an expert for a site visit [16,30]. Despite being one of the world's most populated countries [31], Bangladesh lags many of its South Asian peers in smartphone usage, with only 41 % of mobile phone users owning a smartphone [32]. As of January 2021, there were 171.85 million mobile phone customers [33], and mobile phones have become ubiquitous in practically every home [34]. By the end of July 2021, there were 123.74 million internet users, with 113.69 million accessing the internet through mobile devices [32]. Modern technologies, including smartphone apps, hold promise for optimizing resource usage and reducing human labor in various aspects of life.

With the intensification of aquaculture to boost productivity within a brief time period, there have been resulting issues such as disease outbreaks, water quality problems, and a need for innovative expert advice to solve these emerging problems [35]. Smartphone apps offer significant advantages, allowing farmers to access advice on modern fish domestication techniques and information about the competitive market [36]. Despite the increasing use of digital technologies, smartphones, and the IoT, there has been a limited amount of research conducted on their use in the aquaculture sector in Bangladesh. A few isolated studies explored the application of smartphone apps for fish and shrimp farming [37]. Conversely, a couple of studies have documented the adoption of smartphone apps in the aquaculture sector [38,39]. Recognizing the importance and the existing gap in the adoption of smartphone applications in aquaculture, this study aims to explore the current status of smartphone apps, categorize the different types of apps used in various aquaculture operations, identify the driving forces behind the use of these apps, assess farmers' levels of satisfaction, and evaluate the future potential of using apps in the aquaculture sector in Bangladesh.

## 2 Materials and methods

### 2.1 Study area

**Fig. 1:**
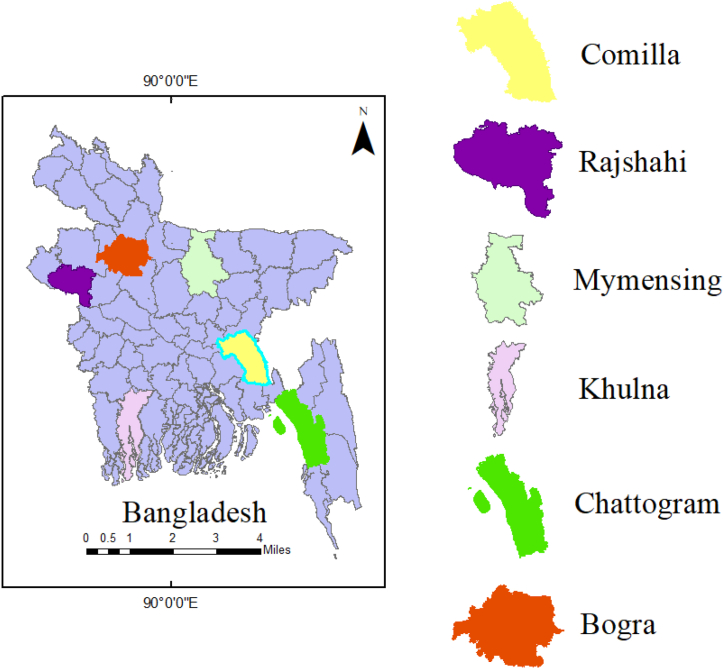
Map showing study area.

Six aquaculture hotspot regions in Bangladesh were selected based on annual fish production data [8]. These regions are Mymensingh, Bogra, Comilla, Chattogram, Rajshahi, and Khulna (Fig. 1). They are geographically representative of fish production in Bangladesh and possess favorable biophysical resources and climatic conditions for aquaculture operations.

### 2.2 Data collection

Both qualitative and quantitative research methodologies were employed to collect data from field surveys and the internet. In field surveys, the data were collected from six aquaculture hotspot regions in Bangladesh from January to December 2021, involving a total of 180 participants. The participants in this study included fish farmers (25 from each district) and aquaculture pharmaceutical traders (5 from each district). Primary field surveys data were obtained through focus group discussions (FGDs) and semi-structured questionnaire interviews. The FGDs were designed to gather a wide range of information simultaneously. A total of 6 FGDs were conducted in the six study sites, each with 10 participants. Random sampling was employed to select farmers and traders for face-to-face questionnaire interviews. Finally, a total of 120 participants were individually interviewed with a semi-structured questionnaire. The aquaculture pharmaceuticals traders were involved to trace the locations of the local farmers, cross-check the data gathered from the farmers, improve data understanding, and remove outliers.

Internet-obtained data were utilized in the compilation of several types of smartphone apps in the aquaculture sector, aiming to enhance the sample size and representativeness of non-probabilistic samples. The primary data collection tools were the Google search engine and the Google Play Store. Due to lack of i-phone users in Bangladesh among the fishers, we did not incorporate the i-store. The keyword typology method was employed to gather data from the source, using search terms such as “apps in fisheries/aquaculture”, “apps for fishers/men/women”, “mobile apps by fishers/men/women”, “apps utilized in fish farms”, “mach chash”, “fish disease”, “biofloc”, and “fishery apps in Bangladesh”. RAS and IMTA are the trending aquaculture system, that's why these keywords also searched in all platform. But no app was found. The sampling method employed in this investigation is Virtual Product Snowball Sampling, resulting in the logging of relevant apps and retrieving the information [[40], [41], [42]]. During the search, it was discovered that there were various apps that encompassed mixed poultry, cattle, and aquaculture. They were included, although they were not exclusively for fish. The focus content of the apps was gathered from the app developers' websites and individual studies of each app. The list of retrieved apps and their features was provided to 17 expert fisheries professionals to identify any apps not included in the search, and the gathered information was confirmed through this process. For each app, downloads, ratings, space, and the year of publishing were gathered from the app store.

### 2.3 Data analysis

Primary data were rechecked, compiled, accumulated, and analyzed using MS Excel and PAST4Project for data interpretation and Pearson-correlation analysis among the app size, rating and download number, OriginPro 2024 for statistical analysis and graph creation, and ArcGIS 8.0 for mapping the study area. The results were represented through tabular and descriptive statistical techniques.

## 3 Results

### 3.1 Present status of smartphone apps used in aquaculture

**Fig. 2:**
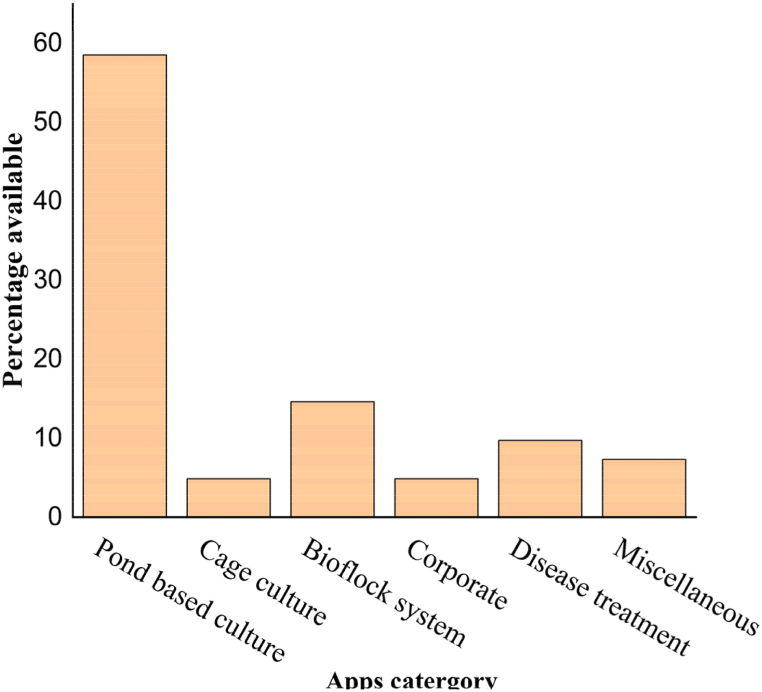
Smartphone apps used in the aquaculture sector of Bangladesh.

A total of 41 smartphone apps related to aquaculture operations were identified in a comprehensive online survey conducted in Bangladesh. The majority (58.53 %) of these smartphone apps were found to be related to pond-based aquaculture. The second-most-found apps (14.63 %) are associated with the biofloc culture system, while aquaculture in cages and corporate apps each covered around 4.87 % individually. Notably, fish disease and treatment-oriented apps covered around 9.75 % of the total. Three applications were identified with various purposes for fish farming, offering guidance on fish breeding and market trends, and connecting fish farmers with potential buyers, collectively covering 7.31 % (Fig. 2). The rest of the apps in the category of miscellaneous deal with different aquaculture tools, i.e., blowers, aerators, and other oxygen enhancers.

### 3.2 Apps related to pond-based aquaculture

The ‘Shrimp Farming BD’ app, developed by Code Tree BD, stands out for its extensive coverage of shrimp and prawn farming details. It incorporates features such as a calculator for practical utility in shrimp and prawn culture, and over 5000 people have downloaded this app from the Google Play Store (Table 1). On the broader spectrum, the “Motso Poramorsho (Fish Advice)" app developed by Reza and Reza Solution provides a holistic view of fish farming, emphasizing disease prevention and treatment, while the “Adhunik Poddhotite Mach Chash” app developed by IT Solution takes a modern approach, covering diverse techniques that downloaded over 10,000 people in Bangladesh (Table 1). The apps “Motso Chashi School” and “Tilapia Mach Chash Poddhoti,” developed by Infotake and Future App Ltd., focused on basic aquaculture learning, employment, and specific fish species, especially Tilapia culture. “Adhonik Poddhotite Deshio Motso Chash” covers various fish species culture and legal aspects, offering a holistic perspective, and it has been downloaded over 1000 times (Table 1). The “Thai Koi Macher Chasher Poddhoti” app specializes in Thai Koi (*Anabas testudineus*) farming, providing practical tools for farmer. “Monosex Golda Chingri Chash” caters to prawn enthusiasts, while “Mach Chash Poddhoti o Kolakoushal” and “Pangas Macher Chasher Poddhoti” offer versatile information. The “Krishi Tottho” app takes a broader agricultural approach. It has been seen that “Adhunik Poddhotite Mach Chash’’, “Motso Chashi School,” “Misro Macher Chash, “Mach Chash-Sohoj Poddhotite Mach Chash,” an “Krishi Shikkha-Mach Chash_Poshu Pakhi Palon” have been downloaded over 10,000 times in Bangladesh. Most of the apps are moderately satisfied by respondents with their services, but there are three apps that are highly satisfied by farmers: “Adhunik Poddhotite Mach Chash,” “Mach Chash-Sohoj Poddhotite Mach Chash,” and “Krishi Shikkha-Mach Chash-Poshu Pakhi Palon” (Table 1). Details function along with all of the app screenshots are shown in Table S1 and Fig. S1 respectively.

**Table 1:** Smartphone apps related to pond-based Aquaculture in Bangladesh.

Sl. no	Apps name	Developer/authority	Size in MB	Rating (Out of 5)	Downloads	Released on	Review
1.	Motsho Chasi Barta	Reza and Reza Solution	13.18	4.4	500 +	Jun 23, 2021	MS
2.	Adhunik Poddhotite mach chash	IT Solution	6.37	4.6	10,000+	Aug 19, 2019	HS
3.	Motso Chashi School	Infotake	5.53	4.3	10,000 +	October 20, 2018	MHS
4.	Tilapia Mach Chash Poddhoti	Future Apps Ltd.	6.61	No	1,000 +	August 8, 2020	MHS
5.	Pangus o Misro mach chash	Super mobile apps	36.80	No	100+	July 6, 2021	LS
6.	Misro Macher chash	Bangla public library	4.08	3.6	10,000+	May 10, 2018	MS
7.	Adhonik poddhotite deshio motso chash	Siraja app store	3.45	No	1000+	December 6, 2019	S
8.	Shing Magur O Koi Mach Chash Poddhoti	Super mobile apps	36.78	No	100+	January 25, 2021	LS
9.	Mach Chash – Sohoj Poddhotite Mach Chash	BD apps station	5.53	4.3	10,000+	June 22, 2018	HS
10.	Lavjonok Poddhotite Mach Chash Poddhoti	Premium Apps Gallery	2.51	4.3	5,000+	September 3, 2019	MHS
11.	Krishi Shikkha -Mach Chash_Poshu Pakhi Palon	Tayra apps studio	3.59	4.3	10,000+	March 21, 2017	HS
12.	Bivinno Jater Mach Chash	Apps star	5.13	4.6	10,000+	August 7, 2017	S
13.	Pukure Korun Shing Macher Chash	Neoapps	2.53	No	1000+	April 13, 2029	S
14.	Motso Poramorsho O Chash Poddhoti	Monju	22.41	5.0	500+	September 29, 2021	LS
15.	Pangas macher chasher poddhoti	Bangla public library	3.98	4.2	5000+	May 10, 2018	MHS
16.	Mach chash poddhoti pukure mach palon	Personal Guide	3.03	4.1	10,000+	January 25, 2019	MHS
17.	Krishi tottho	Radisson Digital Technologies Limited	7.30	4.6	5000+	February 7, 2021	MHS
18.	Thai Koi macher chasher poddhoti	Bangla public library	4.07	4.6	1000+	May 10, 2018	LS
19.	Monosex Golda Chingri chash	Bangla public library	4.0	No	1000+	May 10, 2018	S
20.	Mach chasher poddhoti o kolakoushal	Future Apps Ltd	7.04	4.2	1000+	July 9, 2020	S
21.	Tilapia mach chash ebong niyom	Super mobile apps	36.71	No	100+	June 22, 2021	LS
22.	Catfish Farming	Shahinur Rahman Shajeeb	2.62	No	1000+	December 17, 2021	S
23.	Chingri-Biggan Sommoto Chasher Khutinati	ICAR CIBA	20.23	5.0	1000+	January 21, 2019	S
24.	Shrimp Farming BD	Code tree BD	14.0	4.5	5000+	September 22, 2021	MHS

(N.B: MB = Megabyte, LS = Less satisfied, S = Satisfied, MHS = Moderately high satisfied, HS = Highly satisfied).

### 3.3 Biofloc technology related apps

There were six apps found in the Google Play Store that related to bioflock technology. Based on respondent reviews, these apps are not so popular among farmers; only one app, “Mach chashe notun projukti bioflock,” has been moderately satisfied with 5000+ downloads (Table 2). “Bioflock Poddhotite Mach Chash” offers a comprehensive guide, covering topics from fish farming guidance to recirculating aquaculture systems, with a notable emphasis on water quality management. “Bioflocker Khutinati” provides an in-depth exploration of biofloc equipment, stocking density, and disease management, striking a balance between advantages and disadvantages. “Biofloc BD” stands out for its detailed breakdown of equipment components and meticulous coverage of water quality parameters. “Biofloc Fish Farming” takes a practical approach, focusing on tank preparation, water quality parameter calculations, and quantitative aspects of feed and weight measurements. “Biofloc,” touted as an especially useful app, emphasizes tank capacity and TAN (total ammonia nitrogen) calculations, though a screenshot would aid in assessing its interface. Finally, “Mach Chashe Notun ProjuKti Bioflock” covers a spectrum of topics, from fish species introduction to feed management, with a screenshot offering insight into its user interface. These apps collectively provide a valuable resource for individuals engaged in biofloc fish farming, offering diverse insights and practical guidance. Details of the functions of the biofloc technology-related apps, along with screenshots of all the apps, are shown in Table S2 and Fig. S2, respectively.

**Table 2:** Smartphone apps related to biofloc technology in Bangladesh.

Sl no	Apps name	Developer/authority	Size in MB	Rating (Out of 5)	Downloads	Released on	Review
1.	Bioflock poddhotite mach chash	Apps house production	5.49	5.0	1000+	November 14, 2020	S
2.	Bioflocker khutinati	Creative apps bd	9.47	No	100+	May 13, 2021	LS
3.	Biofloc BD	Abh.masud	11.05	No	100+	April 30, 2021	LS
4.	Bioflog fish farming	Shahinur Rahman Shajeeb	2.45	No	1000+	December 17, 2019	LS
5.	Biofloc	Musa Technologies Ltd	3.92	No	100+	August 5, 2021	LS
6.	Mach chashe notun projukti bioflock	What the app	2.21	4.2	5000+	March 5, 2020	MS

(N.B: MB = Megabyte, LS = Less satisfied, S = Satisfied, MS = Moderately high Satisfied, HS = Highly Satisfied).

### 3.4 Marketing aided corporate apps

Two apps developed by corporate authorities in Bangladesh are specifically dedicated to fish culture and the use of aquaculture pharmaceuticals for commercial aquaculture purposes. The app “Rupali” is a versatile and comprehensive mobile application, providing essential information for commercial aquaculture operations (Fig. S3). This app, developed by ‘Agrow’ on July 25, 2019, has been downloaded over 50,000 times in Bangladesh, with users expressing high satisfaction. Covering topics from fish feed and liming to pond management and modern technology implementation, “Rupali” offers invaluable insights at every stage of aquaculture. Its inclusivity extends to a diverse user base, including farmers, retailers, dealers of feed and aqua chemicals, pond mechanization equipment providers, hatchery owners, fish wholesalers, and officials and researchers from government and non-government organizations. The “Poultry Animals and Aqua Index” or “PAAI” app, developed by “Square informatiX Ltd” on October 3, 2015, serves as a multidimensional platform providing information on poultry and animal products, therapeutic drugs, aqua products, feed additives, and diseases with corresponding treatments. With over 100,000 downloads in Bangladesh, it is highly popular and well-received by users. The app's extensive coverage of common diseases affecting poultry, animals, and aquatic species, along with the inclusion of a screenshot (Fig. S3), enhances its value as a comprehensive guide for disease management in diverse farming operations. Details of the functions of the marketing aided corporate apps are shown in Table S3.

### 3.5 Apps related to fish diseases

There are four apps providing information on fish diseases, diagnosis, measures, and control (Fig. S4). ‘Dr. Fish’ serves as a comprehensive guide, addressing various challenges in fish farming, from water quality management to disease prevention and treatment. The app covers different diseases, their causal agents, and discusses water quality and pond management, making it a valuable tool for fish farmers. ‘Chingrir Rog O Osud’ and ‘Bivinno Macher Rog O Osudh’ focus on diseases in fish and shrimp farming, offering concise overviews of symptoms, causative agents, and preventive measures. ‘Chingri Macher Rog Protikar’ is a gateway to common shrimp diseases, providing valuable advice on prevention and precaution. The app “Bivinno Macher Rog o Osudh” by the Bangla public library has been downloaded over 10,000 times, and users are moderately satisfied with its use (Table 3). Details of the functions of the fish disease-related apps are shown in Table S4.

**Table 3:** Apps related to fish disease in Bangladesh.

Sl. no	Apps name	Developer/authority	Size in Mb	Rating (Out of 5)	Downloads	Released on	Review
1.	Dr. Fish - (Macher Daktar)	Md. Latifur Rahman	27.55	4.7	1000+	April 24, 2019	S
2.	Chingrir Rog Protikar O Protirodh	Super mobile apps	36.49	–	1000+	June 23, 2021	S
3.	Bivinno Macher Rog O Osudh	Bangla public library	4.08	3.5	10,000+	May 10, 2018	MS
4.	Chingri Macher Rog Protikar	Super mobile apps	2.4	4.0	100+	May 10, 2018	LS

(N.B: MB = Megabyte, LS = Less satisfied, S = Satisfied, MS = Moderately high Satisfied, HS = Highly Satisfied).

### 3.6 Apps related to cage aquaculture

Two apps, ‘Khacarvetor Mach Chasher Poddhoti’ and ‘Khacay Mach Chash,’ focus on cage culture techniques (Fig. S5). Developed by the “Bangla public library” in May 2018, ‘Khacarvetor Mach Chasher Poddhoti’ has been downloaded over 1000 times in Bangladesh. On the other hand, ‘Khacay Mach Chash,’ introduced in 2021 by “Super Mobile Apps,” caters to similar needs. Both apps meticulously cover crucial stages such as site selection, water quality, cage building, fish species selection, feed maintenance, and economic aspects related to setting up and managing multiple cages. They offer comprehensive guidance for farmers venturing into cage-based fish farming. Details of the functions of the cage aquaculture-related apps are shown in Table S5.

### 3.7 Miscellaneous apps

The “E-Carp Breeding” app (Fig. S6) stands out by providing extensive information on various carp species, covering natural and artificial breeding techniques, water and soil quality management, seed production, brood fish handling, cost-benefit analysis, and transportation logistics. The “BD Rongin Mach” app is a valuable resource for ornamental fish cultivation, offering insights into distinct species, aquarium equipment, artificial breeding techniques, fry care, disease prevention, and proper oxygen and filter use (Fig. S6). Practical advice on stocking density, water quality, and feeding rules adds value for ornamental fish enthusiasts. The “Mach Chashe Aerator O Blower Bebohar” app is a specialized tool addressing aeration and blower management. It introduces aerators and blowers, explains their use and maintenance, and provides solutions for oxygen-related challenges in ponds. Information on pond filling times and pumping rate equivalents enhances its practical utility for optimizing aquaculture operations. Details of the functions of the miscellaneous apps are shown in Table S6.

### 3.8 Correlation between the size of the apps, ratings, and download number

Table 4 provides the correlation matrix for the variables under study: app ratings, download numbers, and app sizes, utilizing Pearson's correlation coefficients (r) to assess the strength and direction of the relationships. The analysis reveals a weak positive correlation between app ratings and the number of downloads (r = 0.19, *p < 0.01*), indicating that higher-rated apps tend to be downloaded frequently, albeit with a relatively low correlation strength. In case of correlation between the number of downloads and the size of the apps, it found very close to zero (r = 0.025, *p < 0.01*), that does not indicate any kind of directional relationship suggesting there is no tendency for larger or smaller apps to be downloaded. On the other hand, a weak to moderate negative correlation exists between app ratings and app sizes (r = −0.23, *p < 0.05*). The results revealed that while users consider both the ratings and size of apps, ratings play a more influential role in their decision to download an app, as evidenced by the stronger correlation with download numbers.

**Table 4:** Correlation between the size of the apps, ratings, and download number.

Parameters	Rating	Downloads	Size
Rating	**1**		
Downloads	0.19^a^	**1**	
Size	−0.23	0.025^b^	**1**

a Correlation is significant at the 0.01 level (2-tailed).

b Correlation is significant at the 0.05 level (two-tailed).

### 3.9 Awareness among people about smartphone apps

**Fig. 3:**
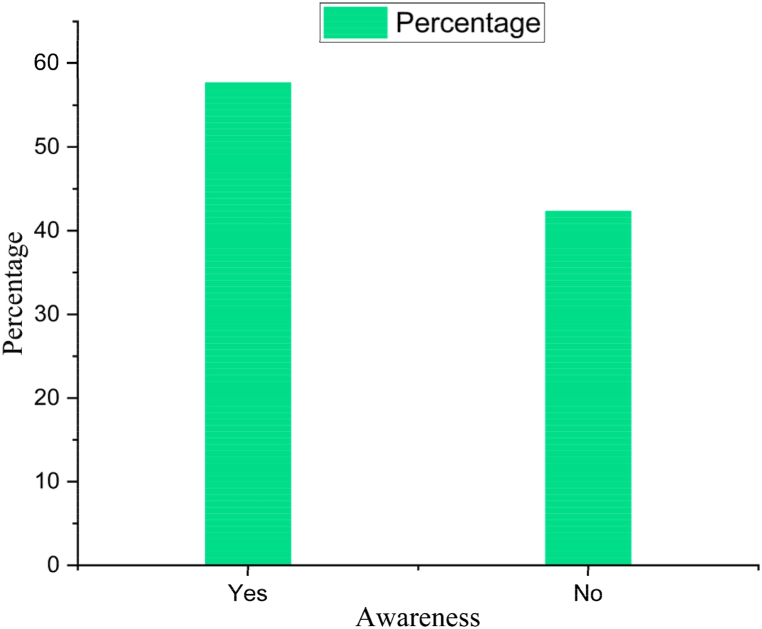
People aware of smartphone apps related to aquaculture.

This research focused on collecting data from six districts that are regarded as aquaculture hotspots in Bangladesh, involving 180 participants. Of these participants, 57.66 % demonstrated awareness of aquaculture-related smartphone apps (Fig. 3), but the rest of the participants had no idea about the aquaculture apps. The study gathered valuable insights, contributing to a nuanced understanding of awareness and utilization of aquaculture apps in these geographic areas.

### 3.10 Identify the driving forces behind the use of these apps

**Fig. 4:**
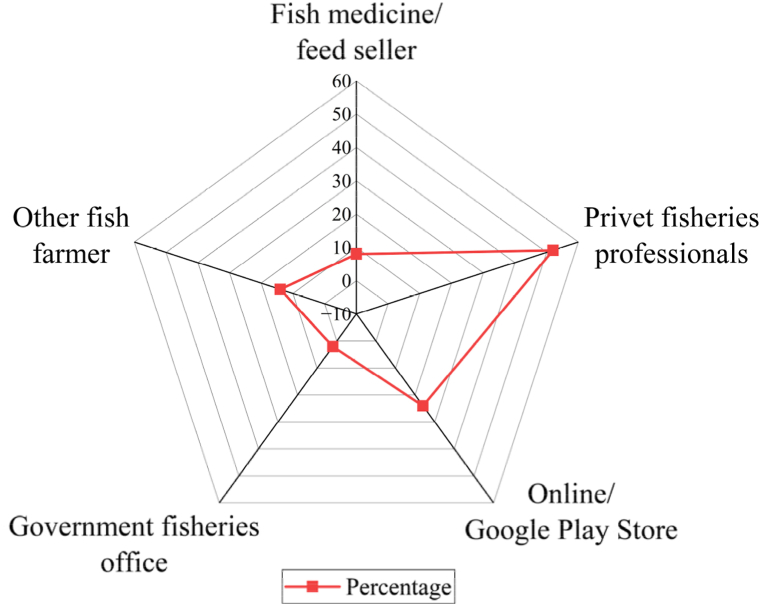
Driving forces behind the use of apps.

The data from the radar diagram (Fig. 4) highlights the pivotal role of private fisheries professionals in disseminating aquaculture-related apps at the field level in Bangladesh. This driving force contributed about 52 % to disseminating the information regarding apps, which indicates that they serve as the primary and most influential source through which individuals become aware of these applications. The second most significant channel through which people discover these apps is through their own mobile app stores, such as the Play Store or Google (24 %). Other local fish farmers also play a vital role (14 %) in sharing information about aquaculture apps, as they find them interesting. Local feed and medicine sellers, being prominent figures due to their long-term experience in the aquaculture sector, also share (8 %) these apps with their customers for better management and production. The data suggests that there is minimal contribution (2 %) from the government fisheries office in disseminating information to fish farmers about these apps.

### 3.11 Level of satisfaction of farmers with using apps

**Fig. 5:**
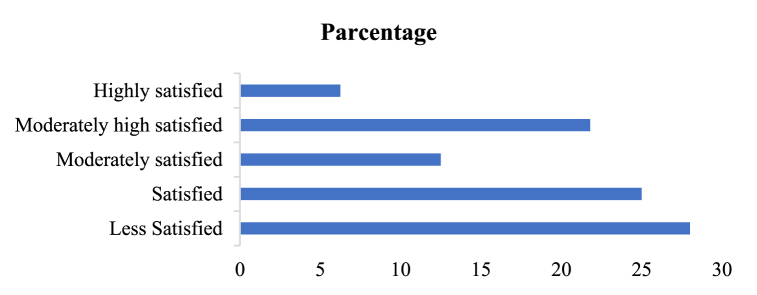
Satisfaction with using apps.

User satisfaction with smartphone apps is intricately tied to how effectively these applications serve people's needs. In today's dynamic digital landscape, the satisfaction level is intricately linked to the apps' ability to provide timely, relevant information and maintain an elevated level of currency through regular updates. This discussion explores the fundamental relationship between user satisfaction, app functionality, and the importance of prompt and pertinent information delivery. We evaluated only the farmers satisfaction in this section because apps are made for farmers. The satisfaction data obtain from 120 farmers among the six locations. We used pharmaceutical traders for cross-check data to remove outliers and improve data quality. Fig. 5 demonstrates that most farmers, specifically around 21.8 %, hold a moderately high level of satisfaction regarding the use of apps in their aquaculture practices. Additionally, approximately 6.25 % of farmers express a higher degree of satisfaction with the applications and their integration into their aquaculture ventures. The data also reveals that approximately 28.012 % of the farmers exhibit a notable level of dissatisfaction with the use of these apps in their aquaculture operations, while 25 % are satisfied with the use of apps.

## 4 Discussion

Advancements in technology, particularly smartphone apps, have significantly increased the efficiency of fish farmers, reduced the costs associated with farming, and minimized human effort in various aspects of aquaculture operations [43]. To elevate aquaculture to a world-class industry, smartphone apps are emerging as a crucial technology. The number of smartphone apps is increasing day by day, and they are poised to become a substantial source of revenue.

In the current study, 41 smartphone apps related to aquaculture were identified for use in Bangladesh, with 24 specifically designed for pond-based aquaculture in Bangladesh. A similar survey study found 124 different smartphone apps available for the fishing industry [44]. However, no apps were found that work with marine fisheries. The current study also found that there were fewer apps related to aquarium fish culture available. This might be due to the lower number of aquarium keepers in Bangladesh [44]. A popular application, ‘AkuaHub,’ provided real images of aquaculture sites and their products, included the sites' latitude and longitude, indexed aquaculture companies, and identified Halal-certified aquaculture companies [45]. In the current study, it was noted that similar mobile apps with these features are lacking in Bangladesh, and there was no evidence of certification for such apps in the country. To enhance the effectiveness of these apps, it is recommended to include certifications for organic or halal products in Bangladesh, as organic fish farming is increasing recently [46].

Current observation revealed two apps (PAAI and Rupali) specifically related to marketing aid in aquaculture. These apps offer farmers invaluable insights into the value chain of aquaculture inputs in Bangladesh, with PAAI having the most downloads compared to all other apps. These apps reduce the distance between stakeholders and provide innovative expert advice at the right time. Similar findings by Huda et al. (2017) stated that mobile phones have reduced the gap between corporate groups and farmers, allowing farmers to directly communicate with relevant stakeholders to obtain proper information about their products [47].

In the present study we have used keyword typology method to collect information about the existing app is Bangladesh through google play store and google. Because there were no many i-Phone users among the farmers, we did not use i-store for searching the apps. Fish farmers would like to use a mobile app that has information on fish culture, disease management, and water quality management [42]. Farmers are interested in obtaining information on feed management, feed suppliers, and market information from these marketing-aided corporate apps. Smart Mariculture K1000 Application, a mobile-based smart monitoring system designed to assist grouper cultivators in Indonesia, introduced by Ref. [48]. This application functions as the user interface for an existing internet-of-things and sensor network, allowing for the monitoring of water and environmental conditions. The app has shown positive results in aquaculture farm monitoring. However, in Bangladesh, there are currently no such specialized apps dedicated to the aquaculture sector. The present observation suggests that both the government and private companies should develop such applications to advance smart aquaculture practices. This study identified four apps that are specifically related to fish disease diagnosis and treatment guidelines. Mobile applications empower both capture fisheries and aquaculture. Fishers can enhance fishing efficiency, and timely diagnosis of fish diseases through smartphone apps can lead to increased profits in aquaculture [30]. A current study found disease-related apps that contain more download numbers in the Google Play Store, but the quality of these apps was not suitable for effective disease management and control. People download this category of app because they are curious about disease management. However, the existing apps do not provide much support. They need to be updated regularly and should include reliable information. In the present study, 180 respondents came from six aquaculture hotspots in Bangladesh. Over 75 % of these farmers expressed at least some satisfaction with their use of aquaculture apps. Notably, in these hotspots, over half of the farmers are aware of smartphone apps related to aquaculture. Drawing parallels, in Telangana farmers in India, revealing that most were middle-aged, owned small fisheries, and exhibited varied smartphone app usage: 60 % more frequently, 30 % more frequently, and 10 % less frequently [16]. These findings collectively underscore the growing significance of smartphone applications in enhancing agricultural practices across diverse regions.

All the apps we found are free to use, providing information on various techniques related to farming practices, feed management, disease diagnosis, and treatment. To make the smartphone app more comprehensive [16], they added a few features, namely: registration guidelines, modern farming techniques, water quality management, buy and sell options, processing, transportation, value-added products, fisheries statistics, and water resources. However, most users expressed a preference for the app to be freely available and not subject to charges.

According to the findings of the present study, more than half of the farmers (57.66 %) acknowledged the awareness and beneficial effects of using smartphone apps in aquaculture. Among farmers in the hotspot area, 52 % first learned about aquaculture apps from private fisheries professionals, while the remaining sources included the Google Play Store, other fish farmers, and aquaculture input sellers, with minimal involvement from government fishery offices. In a similar context, Kiranmayi et al. (2021) [49], reported that farmers obtain information about related apps from the Internet, Krishi Vigyan Kendra, and officials from the Department of Fisheries (DoF). However, other studies, such as the one conducted by Bhendarkar et al. (2020) [50] in Maharashtra, have reported fish feed companies as a significant source of information. In contrast, Dhenuvakonda et al. (2020) [51], found that feed companies were not a prominent source of information.

According to Tian et al. (2015) [52], factors affecting app ratings include the size of the app, marketing efforts, app category, user requirements, and the complexity of the user interface. However, a study by Finkelstein et al. (2017) [53], found a significant relationship between the quantity of downloads and customer ratings. In the present study, a total of 21 apps were rated, and it was found that 19 apps received ratings between 4 and 5. Pearson correlation analysis indicated that downloads were unaffected by app size but had weak positive correlation with app ratings (r = 0.19). This indicates that people were downloading the apps based on their ratings rather than their size. This might be because the apps with higher ratings have excellent quality. But Kiranmayi et al. (2020), found a weak correlation (−0.15) between customer rating and popularity, as well as size and downloads (−0.01) [16].

The current observation revealed that most apps have minimal public reviews. This is largely because many apps maintain extremely poor quality; that's why most of the farmers were less satisfied with using these apps. The data provided by some apps is not entirely accurate, and the organization of certain apps lacks clear visualization. It appears that some developer companies simply create these apps and release them on the Google Play Store without adequate quality checks. This practice can be detrimental to new farmers; if they are misguided by these apps, they might abandon farming altogether. Therefore, a comprehensive review of existing smartphone apps is necessary. Developers should consult fisheries professionals and relevant specialists before creating any apps related to fisheries. Looking forward, there is a need for more in-depth exploration into the specific needs, challenges, and opportunities within the aquaculture sector to inform the development of tailored apps. Future research could delve into the long-term impact of smartphone apps on the industry, considering their role in fostering sustainable practices, influencing economic outcomes for farmers, and contributing to the overall growth of the aquaculture sector in Bangladesh. Addressing these research gaps will provide a more nuanced understanding of the intricate dynamics between technology adoption and the evolution of the aquaculture industry in the region.

## 5 Conclusions

This study has provided a comprehensive overview of the current status of smartphone apps used in the aquaculture industry in Bangladesh. With the identification of 41 smartphone apps catering to various aspects of aquaculture, ranging from pond-based practices to biofloc technology, marketing-aided corporate apps, fish disease management, and cage aquaculture, the research highlights the diversity and potential of technology in advancing the sector. The findings underscore the positive awareness among farmers regarding the benefits of utilizing smartphone apps in aquaculture practices. Private fisheries professionals emerge as crucial driving forces in disseminating information about these apps, surpassing the involvement of government fishery offices. This insight suggests an opportunity for collaboration between private entities and governmental bodies to enhance awareness and adoption of technology in the aquaculture sector. Notably, the study challenges conventional assumptions through correlation studies, revealing a weak correlation between customer ratings and app popularity. Similarly, app size demonstrates minimal impact on downloads, emphasizing the significance of factors such as functionality and effective marketing. The study recommends a collaborative and iterative approach to app development, involving fish farmers to ensure that the apps meet their specific needs and are developed by fisheries researchers or professionals. Language considerations are essential to facilitate easy understanding and adoption within the fish farming community. The IT consulting firm should consider using image-based fish disease detection, water quality monitoring, and other essential information to enhance farmers' understanding when developing new apps. The Department of Fisheries (DoF) should initiate a certification system for newly launched apps to ensure better quality, as many apps currently provide unrealistic information regarding aquaculture operations. Finally, dissemination of these apps to marginal farmers’ levels should be increased. The study advocates for increased involvement of governmental bodies in promoting technology adoption in aquaculture, presenting an avenue for future research.

## CRediT authorship contribution statement

**Diponkor Adikari:** Writing – review & editing, Writing – original draft, Investigation, Formal analysis. **Fatema Jannat Munny:** Writing – review & editing, Writing – original draft, Resources. **Debasish Pandit:** Writing – review & editing, Writing – original draft, Validation, Methodology. **Md Abu Kawsar:** Writing – review & editing, Writing – original draft, Supervision, Resources, Methodology, Investigation, Formal analysis, Data curation, Conceptualization.

## Data availability statement

Data included in article/supplementary material/referenced in article.

## Declaration of competing interest

The authors declare that they have no known competing financial interests or personal relationships that could have appeared to influence the work reported in this paper.
