# Supplementary material for: Smartphone apps pertaining to aquaculture sector in Bangladesh: Current status and future potentials
Source: Heliyon. 2024 Oct 15;10(20):e39396. doi: 10.1016/j.heliyon.2024.e39396 (PMC11532222; doi:10.1016/j.heliyon.2024.e39396)
Supplement: Multimedia component 1 [file mmc1.docx]

**Supplementary File**

**Tables**

**Table S1:** Smartphone apps related to pond-based Aquaculture in Bangladesh

| **Sl no** | **Apps name** | **Function** |
| --- | --- | --- |
|  | Motsho Chasi Barta | Provide information on various fish culture techniques in ponds, fish feed calculation, harvesting, and management techniques. |
|  | Adhunik Poddhotite Mach Chash | Monosex Tilapia (*Oreochromis niloticus*) breeding and farming techniques; Pangas (*Pangasius hypophthalmus*); Shing (*Heteropneustes fossilis*); and Magur (*Clarias batrachus*) polyculture techniques; shrimp farming techniques in ponds and *Ghers*; water quality management; hatchery management; feed management; fish cum paddy culture; cage culture; packing, storing, and marketing of fish; pearl culture; fisheries act; and mariculture. |
|  | Motso Chashi School | Provide information on fish pre-stocking and post-stocking management, various types of fish farming, a brief introduction of exportable aquatic species and their culture techniques, fish and shrimp disease with a causal agent, associated symptoms and treatment, and some selected fish breeding techniques. |
|  | Tilapia Mach Chash Poddhoti | Various types of culture systems, including Tilapia farming, Tilapia associated disease and treatment, bioflock and cage culture, Tilapia and Magur polyculture, monosex Tilapia culture, Kuchia (*Monopterus cuchia*), Ayre (*Sperata aor*), Bata (*Labeo bata*), Rajputi (*Puntius gonionotus*), Chital (*Chitala chitala),* Mola (*Amblypharyngodon mola*), and Dhela (*Osteobrama cotio*) fish farming methods. |
|  | Pangas O Misro Mach Chash | Pangas farming techniques, site selection, seed harvesting, a brief description of disease occurrence with a causative agent, and treatment. |
|  | Misro Macher Chash | Provide insights on Shing, Magur, and Koi (*Anabus testudineus*) polyculture, as well as feed management, site selection, fish seed management, and water quality management. |
|  | Adhonik Poddhotite Deshio Motso Chash | Tara baim (*Macrognathus aculeatus*) culture, tilapia culture, Magur and Shing polyculture, Thai sarpunti culture, Indian major carp farming method, freshwater prawn culture, Pangas culture, crab culture, fish diseases and treatment, nursery pond management, and feed management are all included in this app. |
|  | Shing Magur O Koi Mach Chash Poddhoti | Provides details on the Shingn (*Heteropneustes fossilis*), Magur, and Koi farming methods. |
|  | Mach Chash-Sohoj Poddhotite Mach-Chash | Indigenous fish farming methods include monoculture, polyculture, fish seed, feed and pond management, Thai Sarpunti farming, Thai Koi farming, and freshwater prawn farming. |
|  | Lavjonok Poddhotite Mach Chash Poddhoti | Criteria for fish farm site selection and pond management, fish culture in small ponds, pond preparation and maintenance, seed management, and commercially important fish culture techniques. |
|  | Krishi Shikkha-Mach Chash-Poshu Pakhi Palon | A brief description of indigenous fish farming, monoculture, polyculture, shrimp farming method, fish seed, feed, and pond management, artificial breeding of Boal (*Wallago attu*), and koi farming, and practices of duck, quail, and apiculture. |
|  | Bivinno Jater Mach Chash | Provide information about Shing, Magur, and Koi polyculture, shrimp farming, pangas, tilapia farming techniques, fish disease and treatment, and cage culture techniques. |
|  | Pukure Korun Shing Macher Chash | Provides information on Shing (*Heteropneustes fossilis*) production, site selection, and management. |
|  | Motso Poramorsho O Chash Poddhoti | Pond preparation, nursery management, the introduction of new culture technologies, seed stock management, and essential fish medicine applicable for aquaculture. |
|  | Pangas Macher Chasher Poddhoti | Introduction of various Pangas species, site selection and pond construction, seed collection and transportation, and feed management. |
|  | Mach Chash Poddhoti Pukure Mach Palon | A brief outline of fish farming, pond management and practice, site selection, and water quality management for sustainable aquaculture. |
|  | Krishi Tottho | This is a farming app that teaches about fish farming, animal husbandry, vegetable farming, fruit farming, and horticulture. |
|  | Thai Koi Macher Chasher Poddhoti | A brief understanding of Thai koi farming techniques, including site selection and pond preparation, feeds, and fertilizer management. |
|  | Monosex Golda Chingri Chash | A brief overview of male and female shrimp identification, feed management, and prawn farming guidance. |
|  | Mach Chasher Poddhoti O Kolakoushal | Gives information on Shing, Magur, and Koi polyculture, Pabda (*Ompok pabo*), Pangas, Carp, Shrimp farming techniques, fish diseases and management, bioflock fish culture system, and constraint analysis of fish culture. |
|  | Tilapia Mach Chash Ebong Niyom | Instructions on Tilapia Fish Farming, Monosex Tilapia Culture, Tilapia Feed Requirements, Pond Preparation, and Management |
|  | Catfish Farming | Details on catfish farming and bioflock techniques. |
|  | Chingri-Biggan Sommoto Chasher Khutinati | Details of shrimp farming include site selection, pond preparation, water, feed, and fertilizer management, and diseases and treatments associated with shrimp farming. |
|  | Shrimp Farming BD | Describe semi-intensive shrimp and prawn farming, equipment’s requirements, and feed management. |

**Table S2: Smartphone Apps related to Biofloc technology in Bangladesh**

| **Sl no** | **Apps name** | **Function** |
| --- | --- | --- |
|  | Bioflock Poddhotite Mach Chash | Bioflock fish farming details, RAS (recirculating aquaculture system), introduction of different bioflock equipment and cultivable fish species, fish disease and management. |
|  | Bioflocker Khutinati | Provide information on bioflock equipment, stocking density in tank, water quality management, diseases, causative agents, and therapeutics. |
|  | Biofloc BD | Focused on providing technical details about Bioflock fish farming target species culture in biofloc, feed and medicine estimates, equipment, tank data analysis, and so on. |
|  | Bioflog Fish Farming | Technical details on bioflock fish farming, water quality control, aerator and tank maintenance, and feed management. |
|  | Biofloc | Detailed description of bioflock equipment, fish species selection for biofloc culture, feed, and probiotic dose calculation. |
|  | Mach Chashe Notun Projukti Bioflock | Fish species selection suitable for biofloc, water quality management, aerator and tank maintenance, and feed management. |

**Table S3: Marketing aided corporate apps**

| **Sl no** | **Apps name** | **Function** |
| --- | --- | --- |
|  | Rupali | Aqua medicine, fish feed management, fertiliser application, liming, water quality testing kits, culture management, general problems emerging in the culture system, disease and treatment, and almost all topics related to fish farming are covered. |
|  | Poultry Animals and Aqua Index | It is a multidimensional app that contains information on therapeutic drugs for poultry, large animals, and aquaculture species. Details of therapeutic drugs, feed additives, and some other medicines for fish health and pond management. Common disease of poultry, large animals, fish, and shrimp, along with the causative agent, associated symptoms, and possible treatment. |

**Table S4: Apps related to fish disease in Bangladesh**

| **Sl no** | **Apps name** | **Function** |
| --- | --- | --- |
|  | Dr. Fish-(Macher Daktar) | Fish disease with causal agent, clinical signs and treatment, water quality management, pond preparation, and pond management for aquaculture. |
|  | Chingrir Rog Protikar O Protirodh | A brief overview of the symptoms, causative agent, diagnosis, and treatment of diseases that typically occur in fish and shrimp farming. |
|  | Bivinno Macher Rog O Osudh | Details about diseases that affect finfish include the causative agent, symptoms, prevention, and control techniques, especially different drugs used for treatment. |
|  | Cingri Macher Rog Protikar | Common shrimp diseases with their causative agents, symptoms, prevention, and control measures. |

**Table S5:** **Apps related to cage Aquaculture**

| **Sl no** | **Apps name** | **Function** |
| --- | --- | --- |
|  | Khacar Vetor Mach Chasher Poddhoti | Includes details on cage culture site selection, water current, depth, and quality control, cage building and management, and introduction of cage culture fish species. |
|  | Khacay Mach Chash | Farmers can learn about cage culture site selection, water current, depth, and quality management, cage preparation and management, suitable fish selection, and a cost-benefit analysis of 50 cages. |

**Table S6: Miscellaneous apps**

| **Sl no** | **Apps name** | **Function** |
| --- | --- | --- |
|  | E-Carp Breeding | Introduction of carp species, carp hatchery management, natural and artificial breeding techniques, water and soil quality requirements, seed production, brood fish management, packing, transportation, and dose calculation for fish breeding. |
|  | BD Rongin Mach | Introduction of ornamental fish species, control of aquarium equipment, artificial breeding of aquarium fish, fish domestication, fish disease, and treatment. |
|  | Mach Chashe Aerator o Blower Bebohar | Aerator and blower introduction, aerator use and management, oxygen budget difficulties and solutions, pumping rate equivalents. |

**Figures**

**Figure S1:** Apps related to pond-based aquaculture


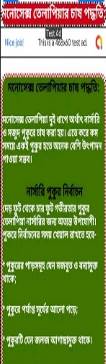

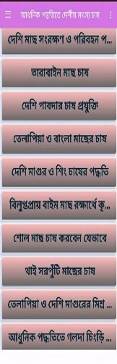

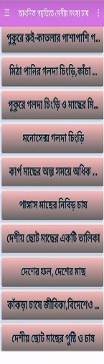

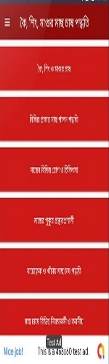

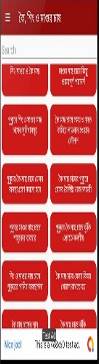

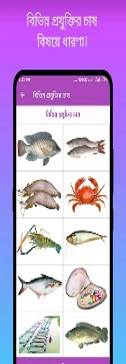

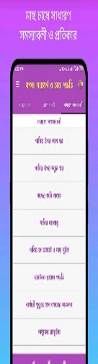

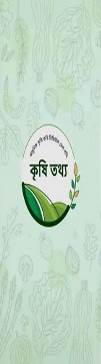

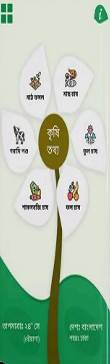

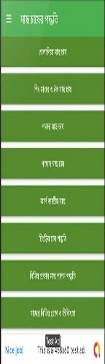

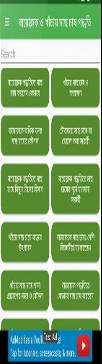

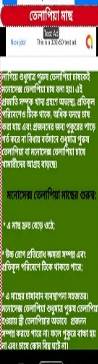


19

20

21

22

23

24


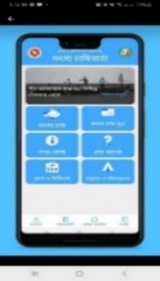

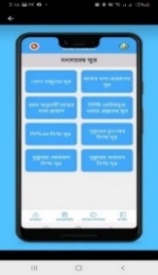

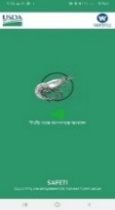

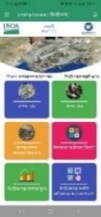

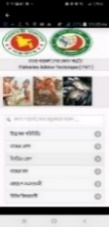

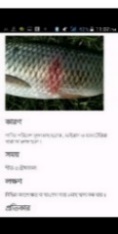

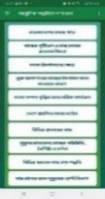

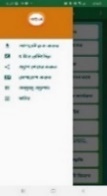

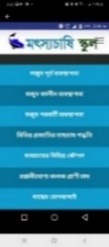

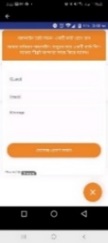

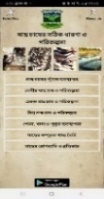

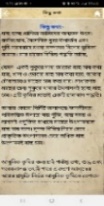

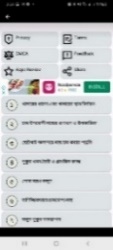

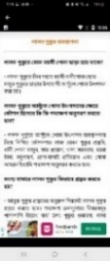

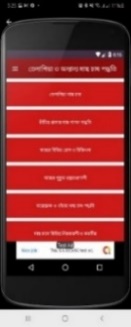

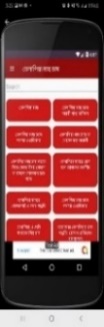

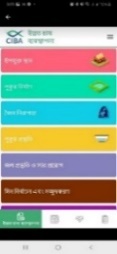

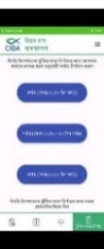

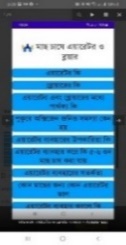

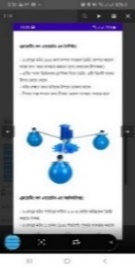

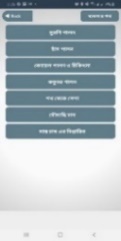

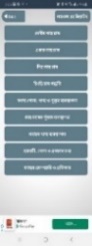

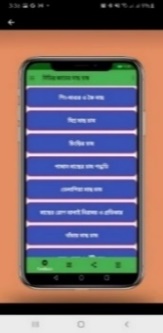

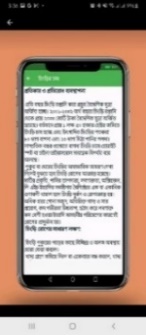

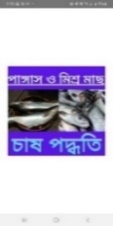

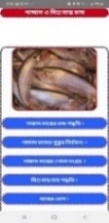

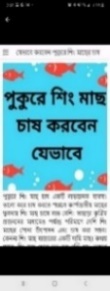

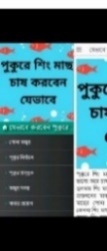

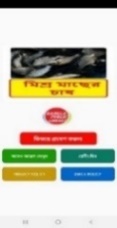

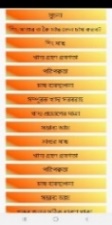

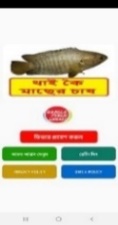

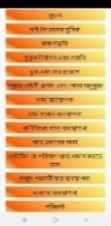

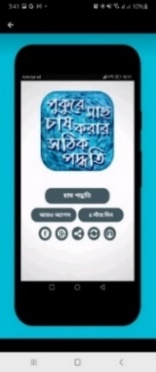

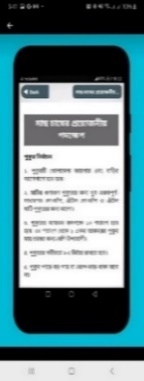

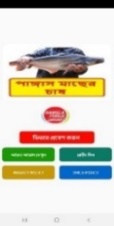

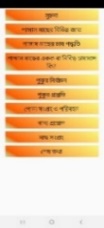


11

2

33

4

5

6

7

85

9

10

11

122222

13

14

15

16

17

18

1

2

3

4

5


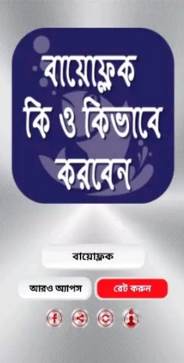

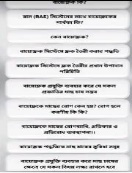

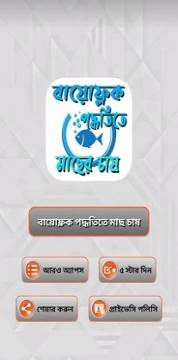

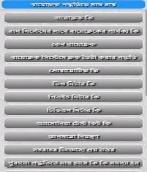

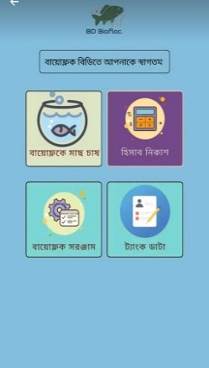

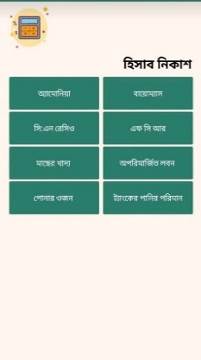

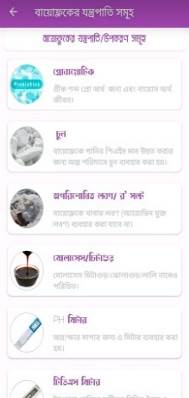

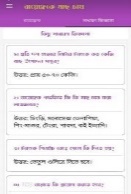

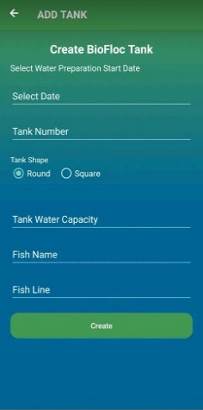

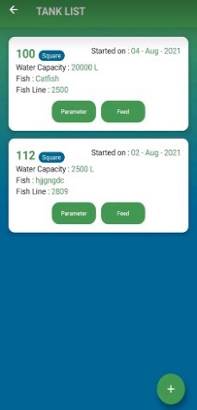

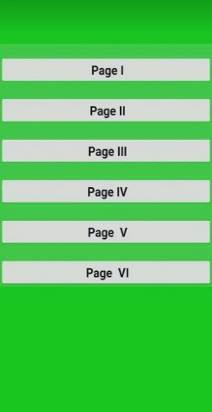

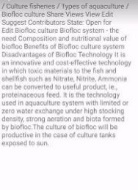


6

**Figure S2**: **Biofloc technology related apps**

**Marketing aided corporate apps**


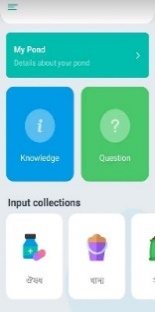

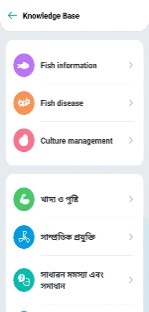

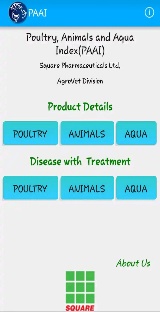

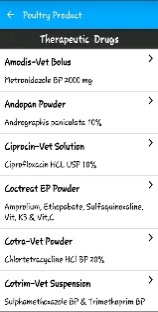


1

2

**Figure S3:** Screenshots of Marketing aided smartphone apps in Bangladesh

(1= Rupali, 2= Poultry Animals and Aqua Index)


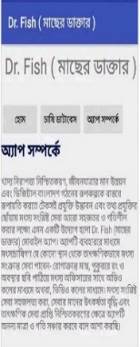

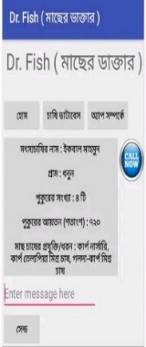

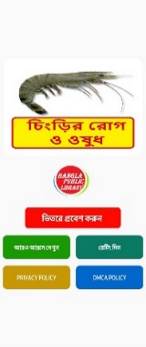

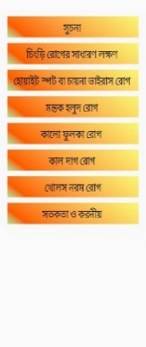

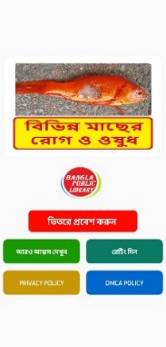

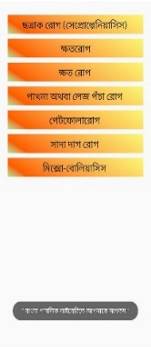

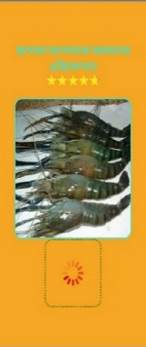

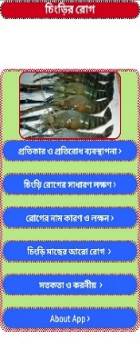


1

2

3

4

**Figure S4:** Screenshots of fish disease related smartphone apps in Bangladesh

(1= Dr. fish, 2= 'Chingrir rog O Osud, 3= Bivinno macher rog o osudh, 4= Cingri macher rog protikar)

**Apps related to cage Aquaculture**


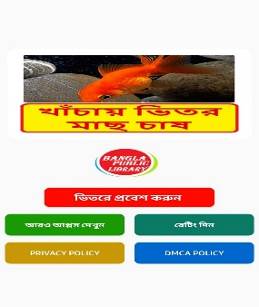

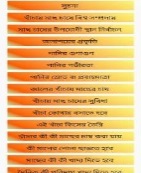

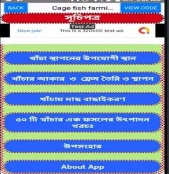

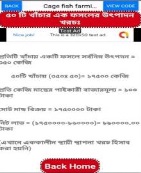


2

1

**Figure S5:** Screenshots of cage aquaculture related apps in Bangladesh

1. Khacarvetor mach casher poddhoti, 2. Khacay mach cash)


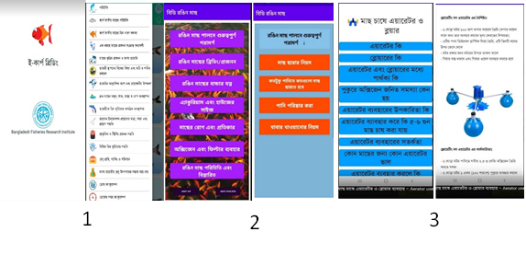
**Miscellaneous apps**

**Figure S6**: Screenshots of miscellaneous apps in Bangladesh

1. E-Carp Breeding, 2. BD Rongin Mach, 3. Mach Cashe Aerator o Blower Bebohar)
