# Supplementary material for: Smartphone apps pertaining to aquaculture sector in Bangladesh: Current status and future potentials
Source: Heliyon. 2024 Oct 15;10(20):e39396. doi: 10.1016/j.heliyon.2024.e39396 (PMC11532222; doi:10.1016/j.heliyon.2024.e39396)
Supplement: Multimedia component 2 [file mmc2.docx]

Age ( Year): ≥

District:

Educational qualification:

1 = No institutional education,

2 = Primary education (1-5 class)

3 = (6-10) S.S.C/H.S.C equivalent

4= Graduation/ Post-graduation/ equivalent

5=PhD

How many days have you been involved in aquaculture/fisheries?

Last 1-3 years

Last 4-7 years

More than 7 years

Do you need any information or advice of aquaculture?

1 = To prevent the diseases of fish

2 = To increase the production
3 = To know about the medicine of fish

Do you know about mobile app ‘FA’?

Has the visit of UFO/DFO/Technical officer reduced by using the smartphone apps?

From where you firstly knew about mobile apps?

1 = DFO/UFO office

2= Fish medicine/feed seller

3= Other fish farmers

4= Private fisheries professionals

From where you firstly collected the mobile apps?

1 = DFO/UFO office

2= Fish medicine/feed seller

3= Other fish farmers

4= Private fisheries professionals

5. Online/ google play store

Have you inspired others to use mobile apps?

If yes, how many numbers of people have been inspired by you?

Have you received any benefits by using the mobile apps ?

1. Yes, 2. No

If yes, what types of benefits have you received

1 = Fish production has

increased

2 = service at doorstep

3 = no cost

4 = immediate service

5 = income has increased

6 = others

Are you satisfied by using this apps?

1= Highly satisfied 2 = Satisfied 3 = Moderately satisfied 4 = Dissatisfied 5= Highly dissatisfied

If dissatisfied, then specify the reasons

Give your suggestions for ensuring the better uses app?
